# Oxidized g-C_3_N_4_/polyaniline nanofiber composite for the selective removal of hexavalent chromium

**DOI:** 10.1038/s41598-017-12850-1

**Published:** 2017-10-09

**Authors:** Rajeev Kumar, M. A. Barakat, F. A. Alseroury

**Affiliations:** 1 Department of Environmental Sciences, Faculty of Meteorology, Environment and Arid Land Agriculture, King Abdulaziz University, Jeddah, 21589 Saudi Arabia; 2 Central Metallurgical R & D Institute, Helwan, 11421 Cairo Egypt; 3 Department of Physics, Faculty of Science, King Abdulaziz University, Jeddah, Saudi Arabia

## Abstract

Nanomaterials with selective adsorption properties are in demand for environmental applications. Herein, acid etching and oxidative decomposition of melon units of graphitic carbon nitride (g-C_3_N_4_) was performed to obtain the oxidized graphitic carbon nitride (Ox-g-C_3_N_4_) nanosheets. Ox- g-C_3_N_4_ nanosheets were further decorated on the polyaniline nanofiber (Ox-g-C_3_N_4_/Pani-NF). Ox-g-C_3_N_4_/Pani-NF was well characterized and further applied for a selective removal of hexavalent chromium (Cr(VI)) form aqueous solution. The zeta potential analysis indicate that the surface of Ox-g-C_3_N_4_/Pani-NF was positively charged which could be beneficial to bind anionic Cr(VI) ions electrostatically. In addition, nitrogen and oxygen containing functional groups exist on the Ox-g-C_3_N_4_/Pani-NF were mainly responsible for adsorption of Cr(VI) ions from aqueous solution. Moreover, the adsorption of Cr(VI) ions was also dependent on solution pH, reaction temperature and initial concentration of Cr(VI) ions. The maximum monolayer adsorption capacity of Ox-g-C_3_N_4_/Pani-NF for Cr(VI), calculated from Langmuir isotherm was 178.57 mg/g at pH = 2 and 30 °C. The activation energy (*Ea* = −20.66 kJ/mol) and the enthalpy change (ΔH° = −22.055 kJ/mol) validate the role of physical forces in adsorption of Cr(VI). These results demonstrate that Ox-g-C_3_N_4_/Pani-NF can be used as a potential adsorbent for environmental remediation applications.

## Introduction

The presence of hexavalent chromium (Cr(VI)) in wastewater is an exigent ecological problem due to its noxious property and amassing in the individual body throughout the food chain^1,2^. To avoid the hazardous effect, Cr(VI) must be removed from wastewater to avoid any possible health and environmental risks. Various methods such as chemical oxidation/reduction, membrane filtration, ion-exchange, and adsorption/sorption have been explored for scavenging of heavy metals from aqueous solution and wastewater^1–9^. Among the various methods, adsorptive separation and solid phase extraction have been considered as a capable technology for confiscation of heavy metals from contaminated water^3,4^. The conventional (activated carbons, polymeric resins, clays) and non-conventional (agricultural and industrial wastes) materials have been reported to remove metallic pollutants from wastewaters^1,3–7^. However, these materials have some intrinsic limitations like low sorption capacity, longer equilibrium time etc^8,9^. Because of these reasons, novel adsorbents with exceptional high adsorption capacities and selective separation are necessary need.

Polymeric graphitic carbon nitride (g-C_3_N_4_) has been explored in environmental remediation, photocatalysis, organic photocatalysis, and in reduction of CO_2_^10–15^. The g-C_3_N_4_ is a low cost ecofriendly two-dimensional conjugated polymer that construct N-bridged “poly(tri-s-triazine)” to form graphitic plane (sp^2^ hybridization) having van der Waals force interaction between the adjacent layers. The g-C_3_N_4_ has multiple defects, good chemical and thermally stable up to 600 °C^11^. One of the major advantages of g-C_3_N_4_ is that its electronic structure is tunable. Few articles published on adsorption properties of g-C_3_N_4_ shows its capacity for the removal of heavy metal. Shen *et al*.^16^ used g-C_3_N_4_ for adsorption of Pb(II), Cu(II), Cd(II) and Ni(II). They observed that the adsorptive separation of metallic pollutants was possible through available nitrogen containing groups. Hu *et al*.^17^ studied the adsorptive removal of aniline and Pb(II) onto the g-C_3_N_4_. The maximum sorption of Pb(II) and aniline onto the g- C_3_N_4_ was possible at pH = 7 and 5. Anbia and Haqshenas^18^ synthesized the functionalized mesoporous g-C_3_N_4_ (surface area = 102.2 m^2^/g) for the adsorptive scavenging of Cu(II) and Pb(II). The maximum amount adsorbed was found to be 199.75 mg/g for Cu(II) and 196.34 mg/g for Pb(II), respectively. Thomas and Sandhyarani^19^ reported the fast adsorption of Cr(VI) onto g-C_3_N_4_-TiO_2_ mesoflowers from aqueous solution.

Various precursors such as melamine, urea, cyanamide and dicyanamide have been applied for the synthesis of g-C_3_N_4_ via a thermal condensation method^16,17,19–21^. Bulk g-C_3_N_4_ has the layered structure which is similar to the graphite. Due to the packed layered structure of g-C_3_N_4_, the active sites between the inter layers do not involve in adsorption process. Some strategies such as thermal chemical etching^21,22^ or ultrasound^23^ methods have applied to exfoliate and modify the layers of g-C_3_N_4_. Niu *et al.* ^21^ reported that Hummers method is not suitable to form nanosheets and bulk g-C_3_N_4_ converts into nanosize particles. Li and coworkers had exfoliated and chemically oxidized bulk g-C_3_N_4_ using a mixture of K_2_Cr_2_O_7_ and H_2_SO_4_. Li and coworkers claimed that a mixture of K_2_Cr_2_O_7_ and H_2_SO_4_ could be efficiently used in exfoliation and oxidized bulk g-C_3_N_4_ into g-C_3_N_4_ nanosheets^24^. However, the complete separation of g-C_3_N_4_ nanosheets from aqueous solution could be challenging due to very small size like the graphene nanosheets, which might be cause nanotoxicity. To overcome this problem, g-C_3_N_4_ nanosheets can be decorated on the surface of other material which not only help in the recovery of g-C_3_N_4_ nanosheets but also enhance its adsorption capacity for metallic pollutants.

Polyaniline (Pani) and its composites have received more attention in few decades because of its easy synthesis, low cost and high adsorption capacity^25,26^. Pani can be easily synthesized by polymerization of aniline in acidic medium and the resulting Pani has net positive charge on its polymeric backbone which can interact electrostatistically with the negatively charged Cr(VI)^27,28^. For instance, Bhaumik *et al*.^27^ synthesized polypyrrole-polyaniline nanofibers adsorbent for removal of Cr(VI) from aqueous solution. Zheng *et al*.^28^ prepared the Pani/Kapok fibers composite adsorbent to remove Cr(VI). By considering an easy synthesis of Pani and adsorption properties, it could be use to develop a new nanocomposite adsorbent with g-C_3_N_4_ nanosheets.

Herein, a ternary mixture of H_2_SO_4_-HNO_3_-H_2_O_2_ is used to exfoliate and oxidative alteration of bulk g-C_3_N_4_ into Ox-g-C_3_N_4_ nanosheets. The obtained Ox-g-C_3_N_4_ nanosheets are then decorated onto the Pani-NF to develop Ox-g-C_3_N_4_/Pani-NF for Cr(VI) removal. Previous studies reported that fibrous Pani has the large surface area compared to normal particles which may be better adsorbent. Thus in this work, the Pani fibers were prepared using soft template method. The crystal structure and chemical states of the synthesized materials have been studied in details. Adsorption studies in brief have further performed for the removal of Cr(VI) from aqueous solution.

## Results and Discussion

**Figure 1:**
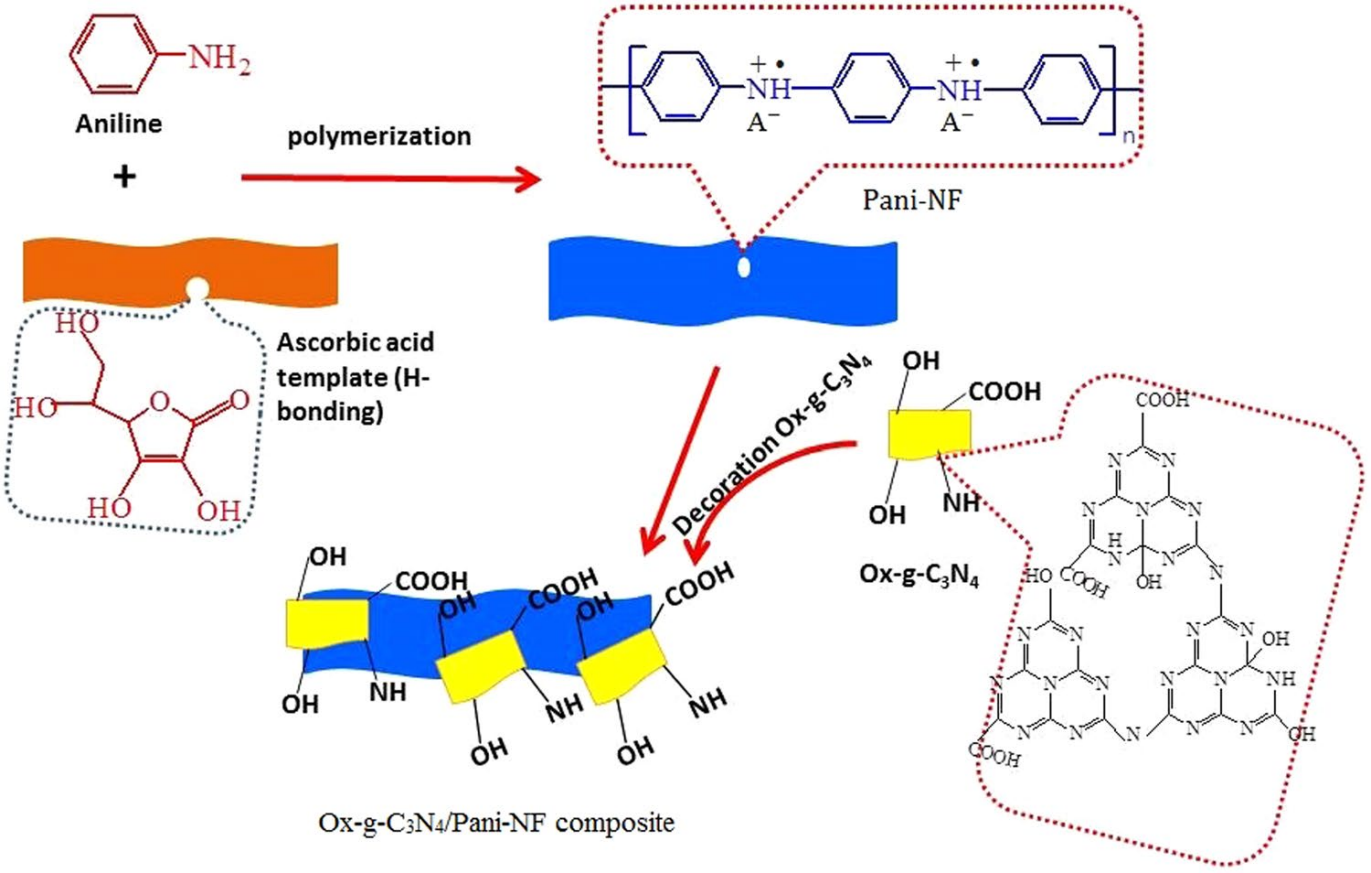
Schematic illustration for Ox-g-C_3_N_4_/Pani-NF composite synthesis.

The strong π−π stacking among sp^2^ carbon atoms is responsible for poor solubility, hydrophobicity and agglomeration of g-C_3_N_4_ nanosheets in various solvents. To overcome this problem, two strategies are applied: (i) introduction of hydrophilic groups on the g-C_3_N_4_ nanosheets and (ii) an auxiliary segregation of hydrophilic g-C_3_N_4_ nanosheets on polyaniline. Acid etching and oxidation process are applied to fabricate highly dispersible hydrophilic g -C_3_N_4_ nanosheets. It is reported in the literature that the oxygen containing functional groups along with defects in the materials can be created effectively using a mixture of strong acid and oxidizing agent at elevated temperature^29,30^. A schematic reaction route for an acid treatment of g-C_3_N_4_ nanosheets (Figure S1) and further synthesis of Ox-g-C_3_N_4_/Pani-NF composite is shown in Fig. 1. In this work, Pani-NF are synthesized through a soft template approach using ascorbic acid. Hydrogen bonding interactions play a vital role in an elongation of Pani nanostructure^31,32^. Ascorbic acid consists four hydroxyl groups that are self-assembled by hydrogen boding interactions and thus, road like structure is formed. The road like self-assembled structure of ascorbic acid helps in the formation of self-assembled Pani-NF^33^. However, ascorbic acid is a reducing agent and hindered the oxidative polymerization Pani-NF. Therefore, polymerization occurs slowly and completed in long time.

**Figure 2:**
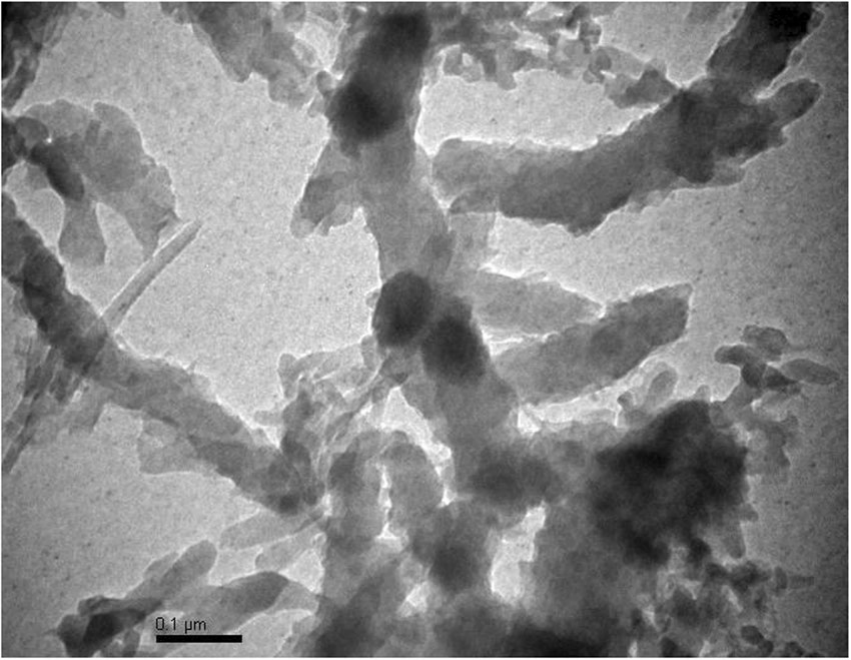
TEM images of Ox-g-C_3_N_4_/Pani-NF composite.

TEM analysis was carried out to observe the morphology of pristine g-C_3_N_4_; Ox-g-C_3_N_4_, Pani-NF and Ox-g-C_3_N_4_/Pani-NF composite. TEM image of bulk g-C_3_N_4_ exhibits solid agglomerates with the size of several micropeters (Figure S2a). It can be visualized from Figure S2b that the interconnected irregular small sheets like particles are obtained after acid-oxidative process. This TEM image confirms a successful reduction in size and alteration of pristine g-C_3_N_4_ nanosheets. Furthermore, a self-assembled ribbon like morphology is appeared for pure Pani powder (Figure S2c). It is also observed that the interconnected small sheets of acid oxidized g-C_3_N_4_ decoration on Pani-NF (Fig. 2), These results are revealing a successful synthesis of Ox-g-C_3_N_4_/Pani-NF composite.

**Figure 3:**
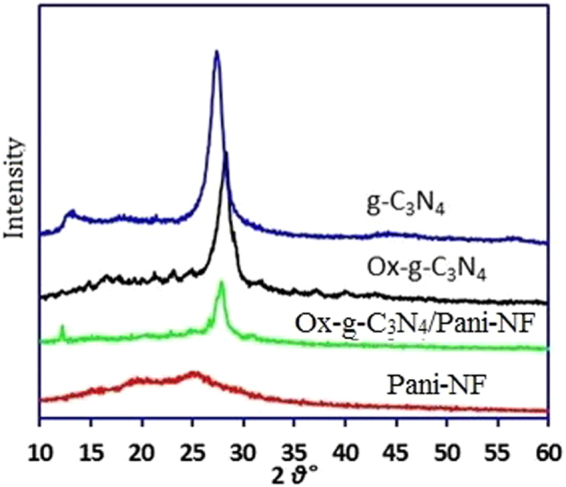
XRD pattern of g-C_3_N_4_, Ox-g-C_3_N_4_, Pani-NF and Ox-g-C_3_N_4_/Pani-NF composite.

The crystal and chemical structure of pristine g-C_3_N_4_, Ox-g-C_3_N_4_, Pani-NF and Ox-g-C_3_N_4_/Pani-NF composite are studied in detail using XRD, XPS and FTIR techniques. The XRD pattern of Pani-NF is presented in Fig. 3. A wide peak ~25° validates an amorphous nature of Pani-NF. The XRD peaks for pristine g-C_3_N_4_ around 12.7° and 27.4°, corresponding to d spacing 0.693 and 0.324 nm are originated due to the interplanar structure packing of motif and carbon nitride interlayer stacking reflections^24^. A slight variation in the XRD pattern of the acid-oxidized g-C_3_N_4_ (Ox-g-C_3_N_4_) nanosheets is observed. The intensity of the peak decreases and its position shifts from 27.42° to 28.2° due to the reduction in the gallery distance between the layers^21,24^. Due to the chemical oxidation and etching, the oxidized g-C_3_N_4_ layers can be planarized by the π−π stacking and H-bonding interactions. These interactions lead to the denser packing and reduction the gallery distance between the layers. The intensity of the XRD peak for Ox-g-C_3_N_4_/Pani-NF composite in compare with pristine g-C_3_N_4_ and Ox-g-C_3_N_4_, is further reduced. This could be because of an interactions between Ox-g-C_3_N_4_ and Pani-NF. In addition, the characteristic peak for Pani is less pronounced in XRD pattern of Ox-g-C_3_N_4_/Pani-NF composite because Ox-g-C_3_N_4_ covered Pani-NF.

**Figure 4:**
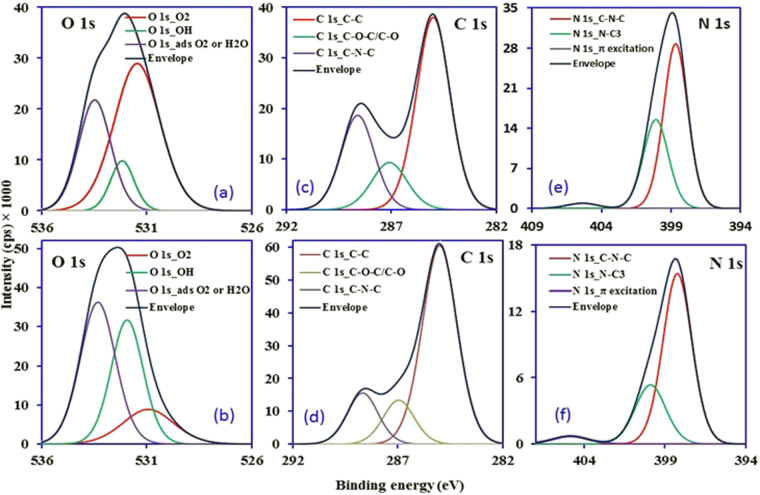
XPS analysis data for Ox-g-C_3_N_4_ and Ox-g-C_3_N_4_/Pani-NF composite, respectively. (**a**,**b**) O 1 s (**c**,**d**) C 1 s and (**e**,**f**) N 1 s.

The introduction of oxygen containing groups in Ox-g-C_3_N_4_ nanosheets after chemical modification is confirmed and analyzed through XPS study and the obtained results are presented in Fig. 4. The atomic percentage obtained from XPS analysis in Ox-g-C_3_N_4_ for O 1 s at 532.06 eV, N1s at 398.86 eV and C1s at 285.01 eV is 21.018, 23.955 and 55.028%, respectively. As shown in Fig. 4a, a strong peak at 532.06 eV for O 1s core level indicates the presence of oxygen containing groups in Ox-g-C_3_N_4_ nanosheets. Three peaks at 531.45, 532.16 and 533.47 eV after deconvolution of O 1 s core level, are detected, which confirm the presence of carboxylic and hydroxyl groups^24,29^. These peaks suggest that the oxygen containing groups are introduced on the surface after chemical treatment of g-C_3_N_4_ nanosheets. A slight variation in peaks position of oxygen species (O 1 s) is appeared at 530.92, 531.93 and 533.31 eV for Ox-g-C_3_N_4_/Pani-NF composite (Fig. 4b). The C1s core level at 285.01 eV is deconvoluted into three main peaks centered at 285, 287.09 and 288.59 eV (Fig. 4c). These are attributed to graphitic sp^2^ C=C bond, C-O bond and sp^2^ hybridized C bonded to N in C-N-C coordination^24,34^. Meanwhile, the peak position of C-C, C-O and C-N-C groups appeared at 285, 286.95 and 288.66 eV does not shows major shift in binding energy of C 1 s core level for Ox-g-C_3_N_4_/Pani-NF composite (Fig. 4d). The N1s spectra (398.86 eV) of Ox-g-C_3_N_4_ also show three different peaks at around 398.68, 400.1 and 405.4 eV after deconvolution (Fig. 4e). These peaks are typically assigned to sp^2^ bonded N atom in C-N=C triazine rings, N-C_3_ bridge atoms and π excitation in C=N or uncondensed terminal amine groups^24,34,35^. Three N 1 s peaks are also obtained in XPS spectrum of Ox-g-C_3_N_4_/Pani-NF composite (Fig. 4f) with slight changes in the peak position at 398.23, 399.89 and 404.85 eV. These results suggest that the chemical states of C, N and O in Ox-g-C_3_N_4_/Pani-NF are similar to Ox-g-C_3_N_4_.

**Figure 5:**
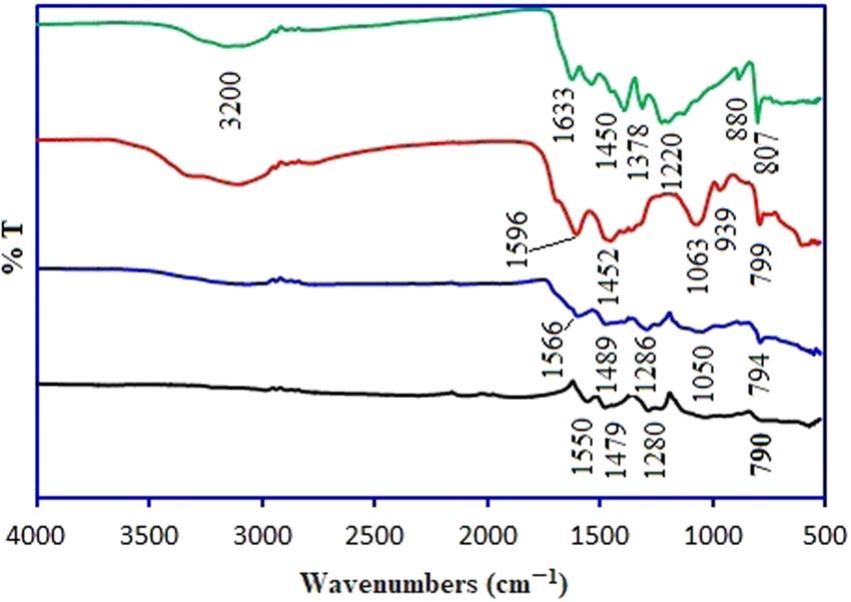
FTIR spectra for pristine g-C_3_N_4_, Ox-g-C_3_N_4_, Pani-NF and Ox-g-C_3_N_4_/Pani-NF composite.

FTIR spectra of pristine g-C_3_N_4_, Ox-g-C_3_N_4_, Pani-NF and Ox-g-C_3_N_4_/Pani-NF composite are shown in Fig. 5. A broad peak ~3000–3400 cm^−1^ for pristine g-C_3_N_4_ nanosheets, is ascribed to the starching vibrations of primary and secondary amine groups. Moreover, broader and sharp peaks are observed for chemically oxidized g-C_3_N_4_ nanosheets. This is due to the introduction of oxygeneous functional groups in modified g-C_3_N_4_ (Ox-g-C_3_N_4_) nanosheets. The adsorption bands at 807 and 880 cm^−1^ are the characteristic peaks for tri-s-triazine units^36^. The peaks at 1220–1450 cm^−1^ are originated due to C-N stretching of aromatic rings and the peak at 1633 cm^−1^ is attributed to the stretching vibrations of C=N^37,38^. After chemical etching, the peaks become more intense and sharp in Ox-g-C_3_N_4_, possibly due to the better-ordered packing of H-bond cohered long stand of polymeric melon units that left after chemical treatment^21^. The peaks at 1063, 1452 and 1596 cm^−1^ in FTIR spectrum of Ox-g-C_3_N_4_ appear due to the presence of C-O, O-H and N-O groups, respectively. However, Larkin *et al*.^39^ reported that skeletal stretching vibrations of C–N and C–O appear in almost same IR regions because of their force constant values. The characteristic absorption bands for Ox-g-C_3_N_4_/Pani-NF composite are similar to Ox-g-C_3_N_4_ and pure Pani-NF with a slight shift in their peak positions and intensities. In Pani-NF spectrum, the characteristic peaks of benzenoid and quinonoid rings occur at 1479 and 1550 cm^−1^. The absorption bands at 1280 cm^−1^ is ascribed to the C-N stretching vibrations^37^. However, these absorption bands are shifted to 1286 cm^−1^ in FTIR spectrum for Ox-g-C_3_N_4_/Pani-NF composite. The characteristic band at 790 cm^−1^ is related to C-H vibration of aromatic ring plane and a slight variation in absorption band from 790 to 794 cm^−1^ for aromatic C-H ring out plane is observed in FTIR spectrum of Ox-g-C_3_N_4_/Pani-NF composite. The significant shift in the characteristic bands of Ox-g-C_3_N_4_ and Pani-NF for Ox-g-C_3_N_4_/Pani-NF composite validate the interfacial interactions between Ox-g-C_3_N_4_ and Pani-NF.

**Figure 6:**
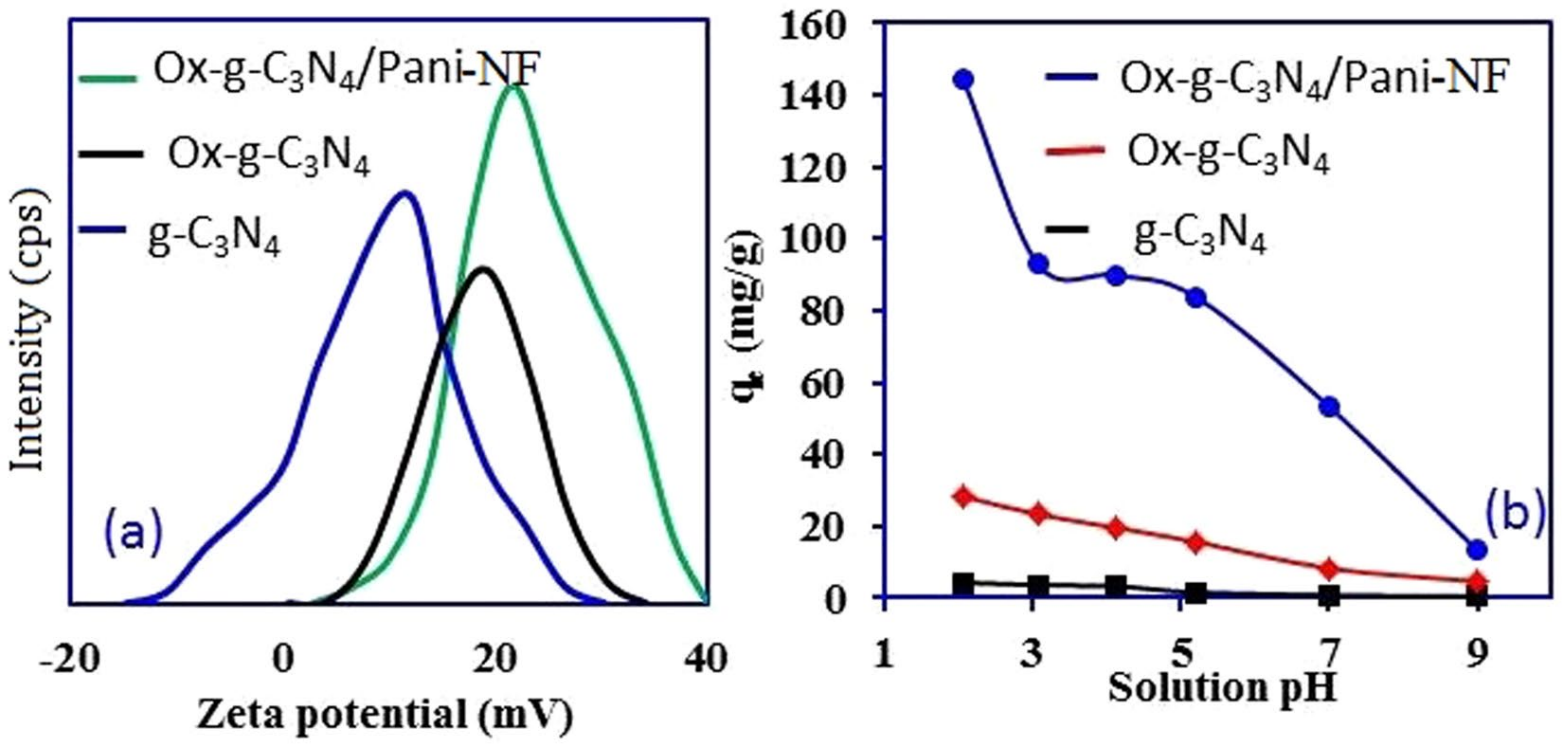
(**a**) Zeta potential and (**b**) effect of solution pH for Cr(VI) adsorption on -C_3_N_4_, Ox-g-C_3_N_4_, Pani-NF and Ox-g-C_3_N_4_/Pani-NF composite.

The surface charge properties of pristine g-C_3_N_4_, Ox-g-C_3_N_4_, and Ox-g-C_3_N_4_/Pani-NF composite were evaluated using a zeta potential analyzer (Malvern, US). The obtained results are shown in Fig. 6a. The zeta potential and surface charge characteristics are increased with alteration in functionality of g-C_3_N_4_ (Fig. 6a). It is reported in the literature that the zeta potential of the stable nanomaterial is close to 30 mV. The zeta potential for Ox-g-C_3_N_4_/Pani-NF composite is found to be +21 mV, which validate its good dispersion and stability in compare with oxidized Ox-g-C_3_N_4_ (+19.2 mV) and pristine g-C_3_N_4_ (11.5 mV)^40^. The positive zeta potential values are attributed to the used of acidic condition for the modification and synthesis of g-C_3_N_4_ and Ox-g-C_3_N_4_/Pani-NF composites, respectively. The carboxyl and hydroxyl groups were created when a strong etching and oxidation of pristine g-C_3_N4 were simultaneously carried out using a ternary mixture of H_2_SO_4_, HNO_3_ and H_2_O_2_. Hence, the net positive charge on the surface of Ox-g-C_3_N_4_ is generated^41^. Similar protocol was used to synthesize Pani-NF and the decoration of Ox-g-C_3_N_4_ nanosheets onto Pani-NF in HCl solution. Amine and imine groups available in the Pani-NF backbone are prone to adsorb H^+^ from aqueous solution. Thus, a highly positively charged Ox-g-C_3_N_4_/Pani-NF composite is obtained. Overall, the synthesized Ox-g-C_3_N_4_/Pani-NF composite has ability to selective binding with the anionic Cr(VI) and a poor binding ability with positively charged Cu(II) owing to its net positive surface charge behavior, (Figure S3). Based on the primary metal adsorption study, Cr(VI) was chosen as a model pollutant to explore adsorption capacity of the synthesized materials.

The effect of adsorbent surface charge and Cr(VI) solution pH on the adsorption process are studied at the varied solution pH in the range from 2 to 9. The results are depicted in Fig. 6b, it can be seen that adsorption of Cr(VI) onto pristine g-C_3_N_4_, Ox-g-C_3_N_4_, and Ox-g-C_3_N_4_/Pani-NF composite increases sharply with decrease in solution pH. The optimum adsorption capacity is attained at pH 2. The solution pH not only influences the surface charge of the adsorbent, but also responsible for the speciation of Cr(VI) in aqueous solution. Cr(VI) exists in various stable forms like H_2_CrO_4_0 HCrO_4_^−^, CrO_4_^2^ and Cr_2_O_7_^2^, which is highly dependent on solution pH. HCrO_4_^−^ is the main species of Cr(VI) at low pH, which can easily bind with the positively charged adsorbent surface though electrostatic interactions^42,43^. The adsorbent surface exhibits an amphoteric behavior with increase in solution pH, because the available functional groups (carboxyl, hydroxyl and amine) on the surface of adsorbents. At pH 1, Cr(VI) exists as H_2_CrO_4_^0^ and HCrO_4_^−^, while at pH 2, Cr(VI) exists mostly as HCrO_4_^−^. The probability of H_2_CrO_4_^0^ adsorption onto positively charged adsorbent surface is low compared to ionic HCrO_4_^−^ due to surface charge. Thus higher Cr(VI) adsorption is expected at pH 2. As the solution pH increases, positive charge on Ox-g-C_3_N_4_/Pani-NF composite surface reduces and the adsorption of Cr(VI) decreases with the increase in solution pH. A net negatively charged surface is developed on the adsorbent which shows an electrostatic repulsion with negatively charged Cr(VI) ions^41^. The adsorption of Cr(VI) on the Ox-g-C_3_N_4_/Pani-NF composite is found to be much higher than the Ox-g-C_3_N_4_ and pristine g-C_3_N_4_ at all the studied pH. This can be attributed to the high positive zeta potential and the large number of surface functional groups (oxygeneous and nitrogenous) present on Ox-g-C_3_N_4_/Pani-NF composite. Because of this reason, Ox-g-C_3_N_4_/Pani-NF composite is further explored for Cr(VI) adsorption at pH = 2.

**Figure 7:**
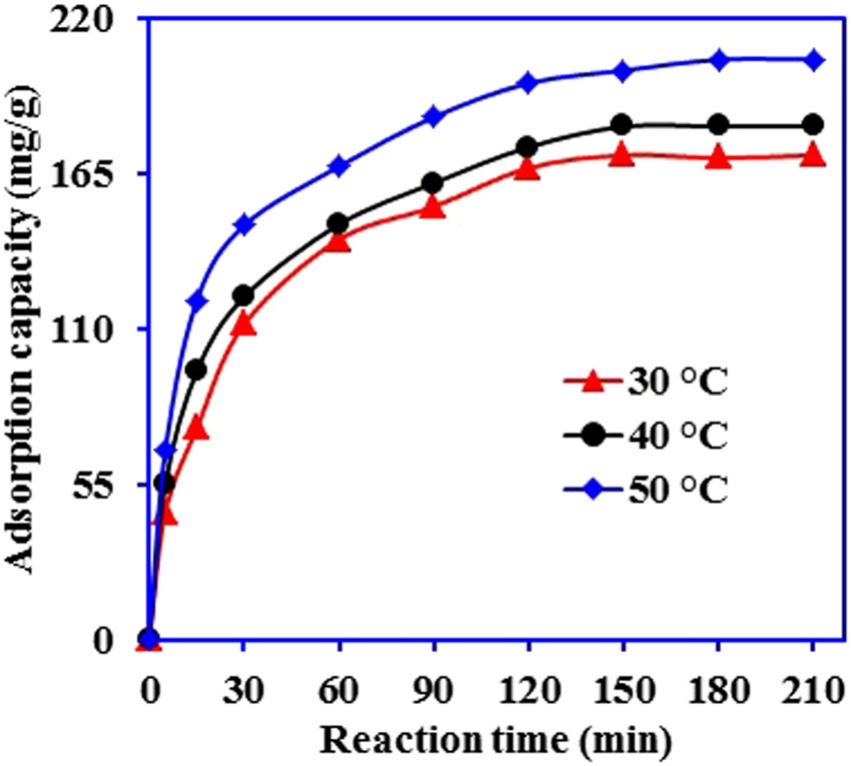
Effect of reaction time and temperature on the removal of Cr(VI) by Ox-g-C_3_N_4_/Pani-NF composite. (conc. -200 mg/L, pH-2.03, Vol. -25 ml, adsorbent mass- 0.015 g).

Figure 7 shows the kinetics of Cr(VI) adsorption on the Ox-g-C_3_N_4_/Pani-NF composite at varied temperature. The adsorption of Cr(VI) increases with the increase in reaction time and equilibrium was established within 150 min. Moreover, reaction temperature also plays a positive impact to alleviate Cr(VI) by Ox-g-C_3_N_4_/Pani-NF composite. The adsorption capacity of Ox-g-C_3_N_4_/Pani-NF composite increases from 174.43 to 205.25 mg/g with increase in solution temperature from 30 to 50 °C, suggesting that adsorption process is endothermic in nature^40^. To confirm the nature of Cr(VI) adsorption onto Ox-g-C_3_N_4_/Pani-NF composite, the data is fitted to the Gibbs and Van’t Hoff equations.

1 $${\rm{\Delta }}{\rm{G}}{\rm{^\circ }}=-{\rm{RT}}\,\mathrm{ln}\,{{\rm{K}}}_{{\rm{c}}}$$

2 $${\rm{l}}{\rm{n}}\,{{\rm{K}}}_{{\rm{c}}}=({\rm{\Delta }}{{\rm{S}}}^{\circ }/R)\,-\,({\rm{\Delta }}{{\rm{H}}}^{\circ }/{\rm{R}}{\rm{T}})$$

where, ΔG°, ΔS° and ΔH° are the free energy change, entropy change, and enthalpy change, respectively. T, K and R are the reaction temperature (K), distribution coefficient and gas constant, (8.314 J/mol k), respectively. The obtained values of ΔG° at 30, 40, and 50 °C are −2.669, −3.054, and −4.307 kJ/mol, indicating the spontaneous nature of adsorption process and the feasibility of Cr(VI) adsorption on the Ox-g-C_3_N_4_/Pani-NF composite^44^. The values of ΔG° ranges from −20 to 0 kJ/mol and −80 to −400 kJ/mol are often for physisorption and chemisorption, respectively^45^. In this study, the obtained ΔG° values indicate that the adsorption of Cr(VI) onto Ox-g-C_3_N_4_/Pani-NF composite is physisorption. The positive value of ΔS (80.988 J/mol k) reflects an increase in randomness at the solid-solution interface via adsorption^46^. Furthermore, the magnitude of ΔH° also reflects an interaction between adsorbent (Ox-g-C_3_N_4_/Pani-NF) and adsorbate (Cr(VI)). The ΔH° for chemisorption is usually between 40 and 120 kJ/mol, while the obtained ΔH° value for Cr(VI) adsorption is 22.055 kJ/mol. Thus, the adsorptive removal of Cr(VI) by Ox-g-C_3_N_4_/Pani-NF composite is due to physisorption^45,47^.

The experimental data presented in Fig. 7 is also fitted to the kinetic models to investigate the mechanism and rate controlling step occurs in Cr(VI) adsorption on the Ox-g-C_3_N_4_/Pani-NF. Pseudo-first order^48^ and pseudo-second order^49^ models are applied and equations of kinetics model, respectively, are:

3 $${\rm{l}}{\rm{o}}{\rm{g}}({{\rm{q}}}_{{\rm{e}}}\,-\,{{\rm{q}}}_{{\rm{t}}})=\,{\rm{l}}{\rm{o}}{\rm{g}}\,{{\rm{q}}}_{{\rm{e}}}\,-\,{k}_{1}{\rm{t}}/2.303$$

4 $${{\rm{t}}/{\rm{q}}}_{{\rm{t}}}=1/{k}_{2}{{{\rm{q}}}_{{\rm{e}}}}^{{\rm{2}}}+{{\rm{t}}/q}_{{\rm{e}}}$$

where q_e_ and q_t_ are the adsorbed amount of Cr(VI) (mg/g) at equilibrium and time t (min). *k*_1_ and *k*_2_ are the pseudo-first order (L/min) and pseudo-second order (g/mg min) rate constants. The plots for the pseudo-first order and pseudo-second order kinetic models are presented in Figure S4a,b and the rate constant values and the calculated equilibrium adsorption capacities, q_e_^cal^ (mg/g), for the pseudo-first order and pseudo-second order kinetic models are tabulated in Table 1. Pseudo-second order model is fitted well to the experimental data than the pseudo-first order kinetic data at all the temperatures studied because of high R^2^ values. The calculated adsorption capacities of Ox-g-C_3_N_4_/Pani-NF composite for Cr(VI) adsorption as predicted from pseudo-second order kinetic model are much closer to the experimental adsorption capacity. This is confirming better fitting of the pseudo-second order kinetic model for adsorption process^50^. Moreover, to find the activation energy (*Ea*) and type of adsorption forces, a linear relationship between the pseudo-second order rate constant (*k*_2_) and temperature (T) is established using Arrhenius equation (5).

5 $$\mathrm{ln}\,{k}_{2}=\,\mathrm{ln}\,{k}_{0}\,-\,(Ea/\mathrm{RT})$$

**Table 1:** Kinetics parameter for adsorption of Cr(VI) onto the Ox-g-C_3_N_4_/Pani-NF composite.

		Pseudo First order model			Pseudo Second order model		
Temp. °C	q_e_^exp^ (mg g^−1^)	q_e_^cal^ (mg g^−1^)	K_1_ (min^−1^)	R^2^	q_e_^cal^ (mg/g)	K_2_ (g/mg min)	R^2^
30	171.431	117.760	2.487 × 10^−2^	0.954	181.185	4.044 × 10^−4^	0.998
40	179.928	111.866	2.303 × 10^−2^	0.960	196.078	3.941 × 10^−4^	0.998
50	205.928	137.911	2.326 × 10^−2^	0.989	217.391	3.265 × 10^−4^	0.999

The magnitude of *Ea* clarifies the forces involved in adsorption. The *Ea* for physisorption varies between 5 to 40 kJ/mol and for chemisorption *Ea* range from 40 to 800 kJ/mol. The *Ea* for Cr(VI) adsorption on the Ox-g-C_3_N_4_/Pani-NF composite is 20.660 kJ/mol, indicating the involvement of physical forces in adsorption process^45^.

**Figure 8:**
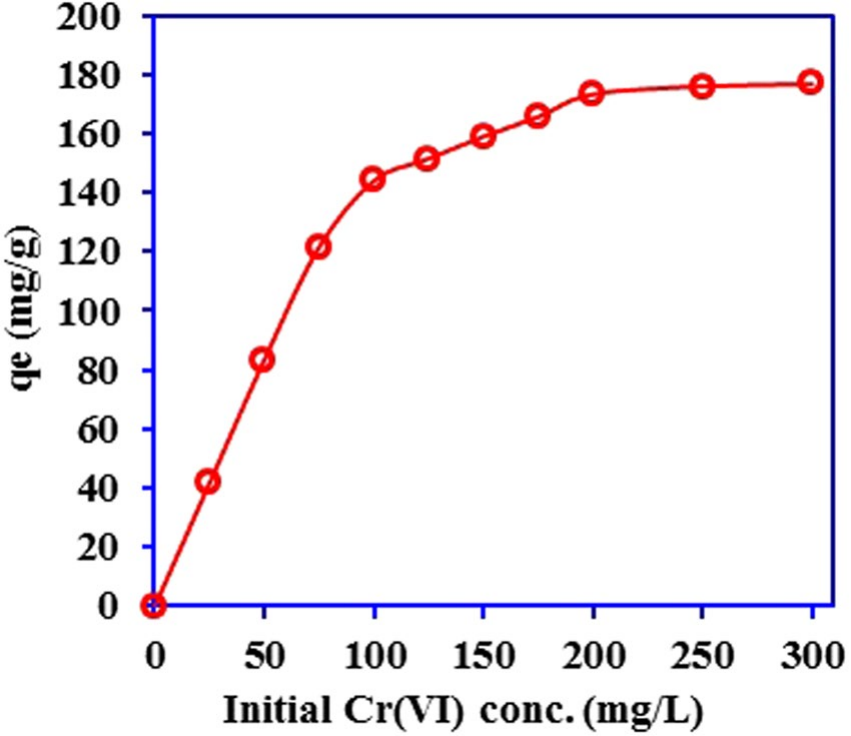
Effect of initial concentration of Cr(VI) on its adsorption onto Ox-g-C_3_N_4_/Pani-NF composite. (time- 210 min, temp. -30 °C, pH-2.03, Vol. -25 ml, adsorbent mass- 0.015 g).

The impact of initial concentrations of Cr(VI) on the adsorption process is studied to find the maximum adsorption capacity and adsorption mechanism for Cr(VI) removal using Ox-g-C_3_N_4_/Pani-NF composite. As depicted in Fig. 8, adsorption capacity increases with initial concentration of Cr(VI) up to 200 mg/L, and thereafter adsorption reached to the plateau due to the saturation of available adsorption sites. The higher possibility of interaction between Cr(VI) and Ox-g-C_3_N_4_/Pani-NF composite at high initial concentration of Cr(VI) is that increase in the mass transfer driving forces^51^. The equilibrium adsorption data presented in Fig. 8 is analyzed using Langmuir and Freundlich isotherm models. The Langmuir isotherm model is based on the monolayer coverage while the Freundlich isotherm model postulates an equilibrium on the heterogeneous adsorbent surface. The Langmuir equation can be represent as:

6 $$({{\rm{C}}}_{{\rm{e}}}{/{\rm{q}}}_{{\rm{e}}})=({{\rm{C}}}_{{\rm{e}}}{/{\rm{q}}}_{{\rm{m}}})+({1/\mathrm{b\; q}}_{{\rm{m}}})$$

where, q_m_ is the maximum monolayer adsorption capacity (mg/g) and C_e_ is the Cr(VI) concentration at equilibrium (mg/L) and b is a constant related to the energy of adsorption (L/mg). q_m_ and b are calculated from the slope and intercept of a linear plot of C_e_/q_e_ vs. C_e_ (Figure S5a).

The Freundlich isotherm model can be represented as:

7 $$\mathrm{ln}\,{{\rm{q}}}_{{\rm{e}}}=(1/{\rm{n}})\mathrm{ln}\,{{\rm{C}}}_{{\rm{e}}}+\,\mathrm{ln}\,{{\rm{K}}}_{{\rm{F}}}$$

where, q_e_ is the adsorption capacity at equilibrium (mg/g), K_F_ and n are constants that stands for the capacity and intensity, respectively. The parameters for Freundlich isotherm model are calculated from a plot of ln q_e_ vs. ln C_e_ (Figure S5b).

The calculated values of the Langmuir isotherm parameters q_m_ and b are 178.57 mg/g and 0.370 L/mg. On the other hand, the values of the Freundlich isotherm parameters n and K_F_, are 5.238 and 72.893 L/mg. It is noted that the correlation coefficient (R^2^) value for the Freundlich isotherm is lower (0.7247) than that for the Langmuir isotherm (R^2^–0.9986). This indicate that the Freundlich isotherm model is not suitable to describe Cr(VI) removal using Ox-g-C_3_N_4_/Pani-NF composite. The Langmuir isotherm model is much fitted well to the adsorption of Cr(VI) by Ox-g-C_3_N_4_/Pani-NF composite. Thus, adsorption behavior of Cr(VI) on the Ox-g-C_3_N_4_/Pani-NF composite seems to be monolayer and the possibility for interactions between adjacent Cr(VI) ions is negligible^51,52^. In addition, an essential feature of the Langmuir isotherm model is in term of dimensionless separation factor (R_L_). For the favorable adsorption of Cr(VI) on the Ox-g-C_3_N_4_/Pani-NF composite, the R_L_ values must be in between 0 and 1. R_L_ > 1 and R_L_ = 0 indicate the unfavorable and irreversible adsorption process, respectively^53^. The R_L_ can be defined as:

8 $${{\rm{R}}}_{{\rm{L}}}=1/(1+{\rm{b}}\,{{\rm{C}}}_{{\rm{0}}})$$

where, C_0_ is initial concentration of Cr(VI) (mg/L) and b is the Langmuir constant (L/mg). The R_L_ values obtained for Cr(VI) adsorption by Ox-g-C_3_N_4_/Pani-NF composite are in the range 0.097 and 0.010, which indicate the favorable adsorption process for Cr(VI), and the suitability of the Langmuir isotherm model for the adsorption equilibrium data.

To find the effectiveness of the synthesized material, the adsorption capacity of Ox-g-C_3_N_4_/Pani-NF composite has been compared with the previously reported adsorbents used for the removal of Cr(VI). The maximum monolayer adsorption capacities of various adsorbents and applied experimental conditions have been reported in Table 2. The results in Table 2 revealed that adsorption capacity of the adsorbents is highly dependent on the experimental conditions and used adsorbent. The adsorption capacity of Ox-g-C_3_N_4_/Pani-NF composite is comparatively higher than the previously reported adsorbents.

**Table 2:** The Maximum adsorption capacity of various adsorbents used for the removal of Cr(VI).

Adsorbent	Adsorption capacity (mg/g)	Experimental conditions						Ref.
		pH	Conc. (mg/L)	Vol. (ml)	Temp. (°C)	Time (h)	Dose (g)	
Ox-g-C_3_N_4_/polyaniline-NF	178.57	2	25–300	25	30	3	0.015	This study
Rice husk	31.1	6	50–200	10	25	48	0.1	1
Polyaniline	122.2	4.5	100–400	25	30	3	0.05	28
Kapok fiber/polyaniline	65.65	4.5	—	25	30	3	0.05	28
Fe_3_O_4_@SiO_2_–mPD/SP	158.73	—	50–275	—	30	24	1.0	42
DBSA-Polyaniline/MWCNTs	55.55	2	20–140	15	30	10	0.02	49
Bamboo charcoal grafted by Cu^2+^-N-aminopropylsilane	17.938		2–12	50	30	4	0.1	53
Amino functionalized GO/Fe_3_O_4_	123.4	2	—	—	20	12	0.2	43
copper-benzenetricarboxylates	48	7	10–40	10	25	—	0.005	54
polyaniline/palygorskite	16.22	5.5	2.5–35	40	35	24	0.02	55
Longan seed activated carbon	169.49	3	50–500	50	25.2	6	0.1	56

## Conclusion

A novel anion selective positively charged Ox-g-C_3_N_4_/Pani-NF composite has been synthesized and characterized using various instrumental techniques. The results are showing a capability of H_2_SO_4_-HNO_3_- H_2_O_2_ to exfoliate, cut and oxidized the bulk g-C_3_N_4_ into oxidized g-C_3_N_4_ nanosheets. TEM image clearly shows an alteration in bulk g-C_3_N_4_ nanosheets. XPS analysis is confirmed the oxidation of bulk g-C_3_N_4_ after chemical modification. The characterization results demonstrate a successful synthesis of multifunctional Ox-g-C_3_N_4_/Pani-NF and its selectivity for adsorption of Cr(VI) from aqueous solution. The adsorption of Cr(VI) significantly increases as the functionality of g-C_3_N_4_ changes as g-C_3_N_4_ < Ox-g-C_3_N_4_ < Ox-g-C_3_N_4_/Pani-NF composite. The optimum adsorption for Cr(VI) using Ox-g-C_3_N_4_/Pani-NF was attained at pH 2 within 180 min. The adsorption capacity of the Ox-g-C_3_N_4_/Pani-NF composite increases with temperature from 30 to 50 °C, revealing the endothermic nature of adsorption process. The Cr(VI) mass transfer rate is well described by pseudo-second order kinetic model. The equilibrium data are well fitted with the Langmuir isotherm model and the obtained values suggest a monolayer adsorption of Cr(VI) on the Ox-g-C_3_N_4_/Pani-NF composite. Based on these observations, Ox-g-C_3_N_4_/Pani-NF composite can be considered as anion selective adsorbent for the separation and removal anionic pollutants present in wastewater.

## Materials and Methods

### Materials

Aniline and oxidant potassium per-sulphate were obtained from BDH Ltd and SD Fine chemical Ltd, respectively. Sulphuric acid (98%), nitric acid (69%) and hydrogen peroxide were purchased from Panreace Qumica S.A.U. Melamine was obtained from Techno Pharmachem Haryana, India. Potassium dichromate, used for the preparation of Cr(VI) solution was provided by BDH chemical Ltd., Poole England.

### Oxidation of g-C_3_N_4_

The g-C_3_N_4_ was prepared by thermal heating of melamine at 550 °C in a muffle furnace for 3 h at the heating rate of 5 °C/min. A yellow powder of g-C_3_N_4_ was obtained and thereafter, 2 g powder was added into 40 ml mixture solution of concentrated H_2_SO_4_ (98%) and HNO_3_ (69%) (1:1). The resulting mixture was heated at 40 °C under sonication for 2 h and 3 ml H_2_O_2_ (33%) was then added dropwise and further sonicated for an additional 3 h for exfoliation. A whitish-yellow product was attained and 150 ml of deionized water was also added into the suspension. The dilute suspension of the oxidized g-C_3_N_4_ (Ox- g-C_3_N_4_) was centrifuged at 10000 rpm and washed alternatively with DI water, acetone and dried in oven at 70 °C for 12 h. Finally, the yellow colour exfoliated Ox-g-C_3_N_4_ sheets were obtained.

### Preparation of Ox-g-C_3_N_4_/polyaniline nanofibers composite

Polyaniline nanofibers (Pani-NF) were initially synthesized by dissolving 0.88 g ascorbic acid in 100 ml HCl solution (1 M) and further stirred for 30 min. Then 1.6 ml aniline was added and stirred until a uniform solution obtained. Thereafter, the resulting solution was cooled in a refrigerator for 2 h and 100 ml of the cooled 0.1 M ammonium persulfate solution was added dropwise under continuous stirring. The polymerization was then allowed to extend transferring the solution in a refrigerator for 24 h without any agitation. The greenish product was obtained which was sonicated for 15 min and then stirred for 1 h. Afterwards, 0.5 g Ox-g-C_3_N_4_ suspended in 100 ml DI water using a sonicator for 2 h was added to polyaniline solution and further stirred for 24 h. The resulting precipitate was filtered, washed with DI water, acetone and dried in oven at 70 °C for 12 h to obtain Ox-g-C_3_N_4_@polyaniline nanofibers (Ox-g-C_3_N_4_/Pani-NF). Pani-NF was also synthesized by adopting a similar method without adding Ox-g-C_3_N_4_.

### Instrumentation

The microstructure of g-C_3_N_4_, Ox-g-C_3_N_4_, Pani-NF, and Ox-g-C_3_N_4_/Pani-NF was examined by transmission electron microscopy (TEM) (model Tecnai G2 F20 Super Twin) at an accelerating voltage of 200 kV. Phase analysis was performed by X-ray diffractometer, Ultima-IV, Rigaku Corporation, Tokyo, Japan using Cu Kα radiation. The Fourier transform infrared (FTIR) spectra for g-C_3_N_4_, Ox-g-C_3_N_4_, Pani-NF, and Ox-g-C_3_N_4_/Pani-NF were recorded over a range of 400–4000 cm^−1^ using the Perkin Elmer Spectrum 100 FTIR Spectrometer. The chemical state and surface composition of Ox-g-C_3_N_4_ and Ox-g-C_3_N_4_/Pani-NF were analysed by X-ray photoelectron spectroscopic (XPS), SPECS GmbH, (Germany) spectrometer, using Mg- Kα(1253.6 eV X-ray source) at 13.5 kV, 150 W X-ray power.

### Adsorption of hexavalent chromium

The adsorption of Cr(VI) was studied by mixing 0.015 g of the synthesized materials into 25 ml solution of metal ions stirring at 200 rpm in dark. The effect of solution pH was examined by varying solution pH in the range from 2 to 10 and the solution pH was adjusted using 0.1 M HCl or NaOH solution. The effect of initial Cr(VI) concentration was investigated at the varied concentrations from 25 to 300 mg/L at 30 °C. The equilibrium time studies were performed in a series of conical flasks agitated in the time range from 5 to 210 min at various temperature 30, 40 and 50 °C. After equilibrium attainment, the adsorbed amount of Cr(VI) by adsorbent was determined using a HACH curette test LCK313 reagent (Total chromium analysis). The adsorption capacity of the adsorbent was calculated in per unit mass of the adsorbent.

The reduction of Cr(VI) into Cr(III) is commonly observed at low pH and in the presence of adsorbent like polyaniline^28^. Although both mechanism, adsorption and reduction of Cr(VI) are difficult to separate. Therefore, total chromium analysis were performed to analyze the remaining amount of the chromium in the solution and the total amount of Cr(VI)/Cr(III) adsorbed considered as Cr(VI) removal on the adsorbent surface.

## Electronic supplementary material

Supplementary Information
