# Supplementary material for: Oxidized g-C3N4/polyaniline nanofiber composite for the selective removal of hexavalent chromium
Source: Sci Rep. 2017 Oct 9;7:12850. doi: 10.1038/s41598-017-12850-1 (PMC5634480; doi:10.1038/s41598-017-12850-1)
Supplement: Supplementary file 1 — Supplementary Information [file 41598_2017_12850_MOESM1_ESM.pdf]

Supporting Information's

Oxidized g-C_3N_4/polyaniline nanofiber composite for the selective removal of hexavalent chromium

Rajeev Kumar[a*], M.A. Barakat[a,b] , F.A. Alseroury[c]

[a]*Department of Environmental Sciences, Faculty of Meteorology, Environment and Arid Land Agriculture, King Abdulaziz University, Jeddah, 21589, Saudi Arabia*

[b]*Central Metallurgical R & D Institute, Helwan 11421, Cairo, Egypt*

[c] *Department of Physics, Faculty of Science, King Abdulaziz University, Saudi Arabia*

**Corresponding author:* olifiaraju@gmail.com

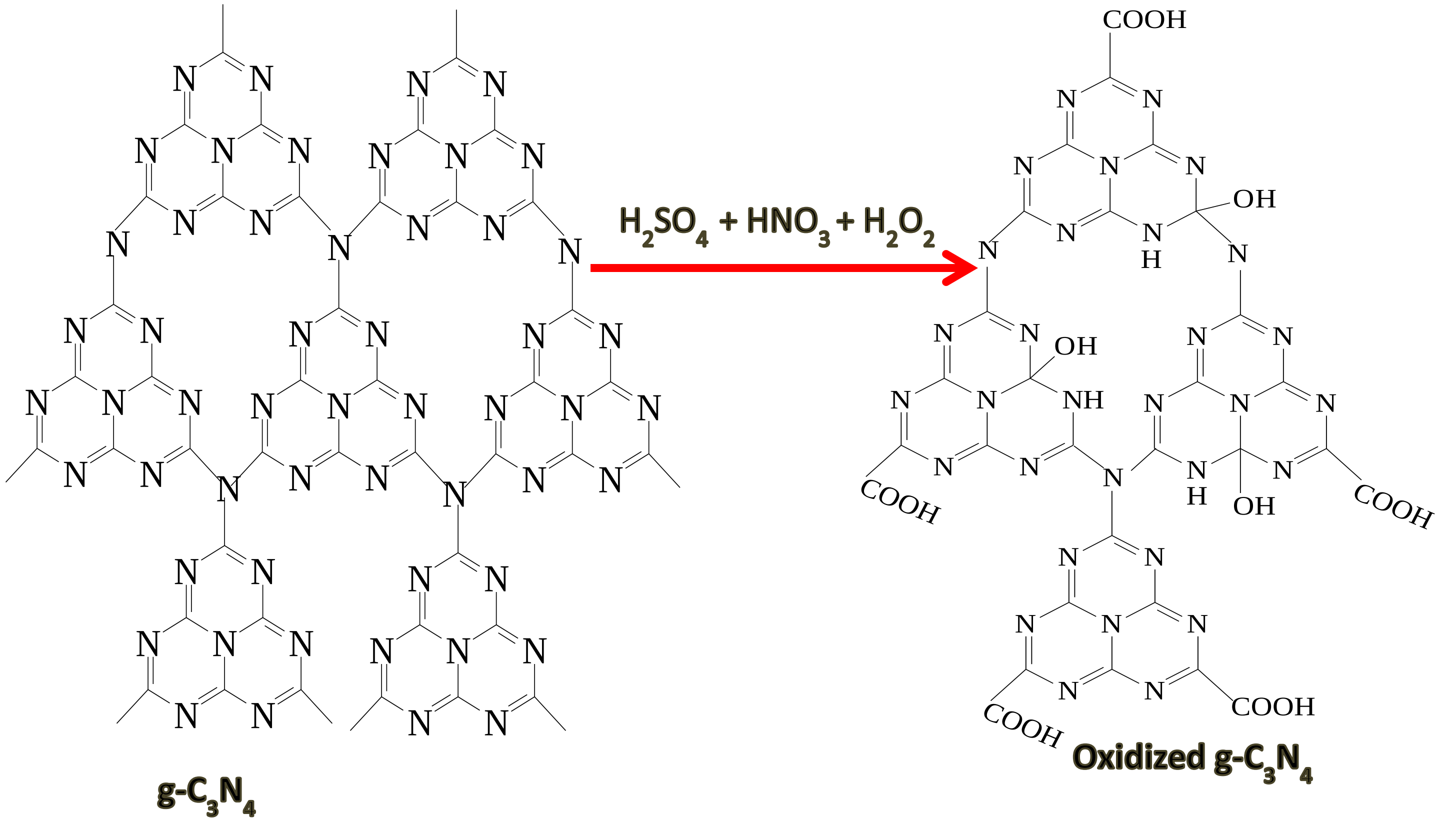

Figure S1. Schematic illustration of chemical oxidation of bulk g-C_3N_4

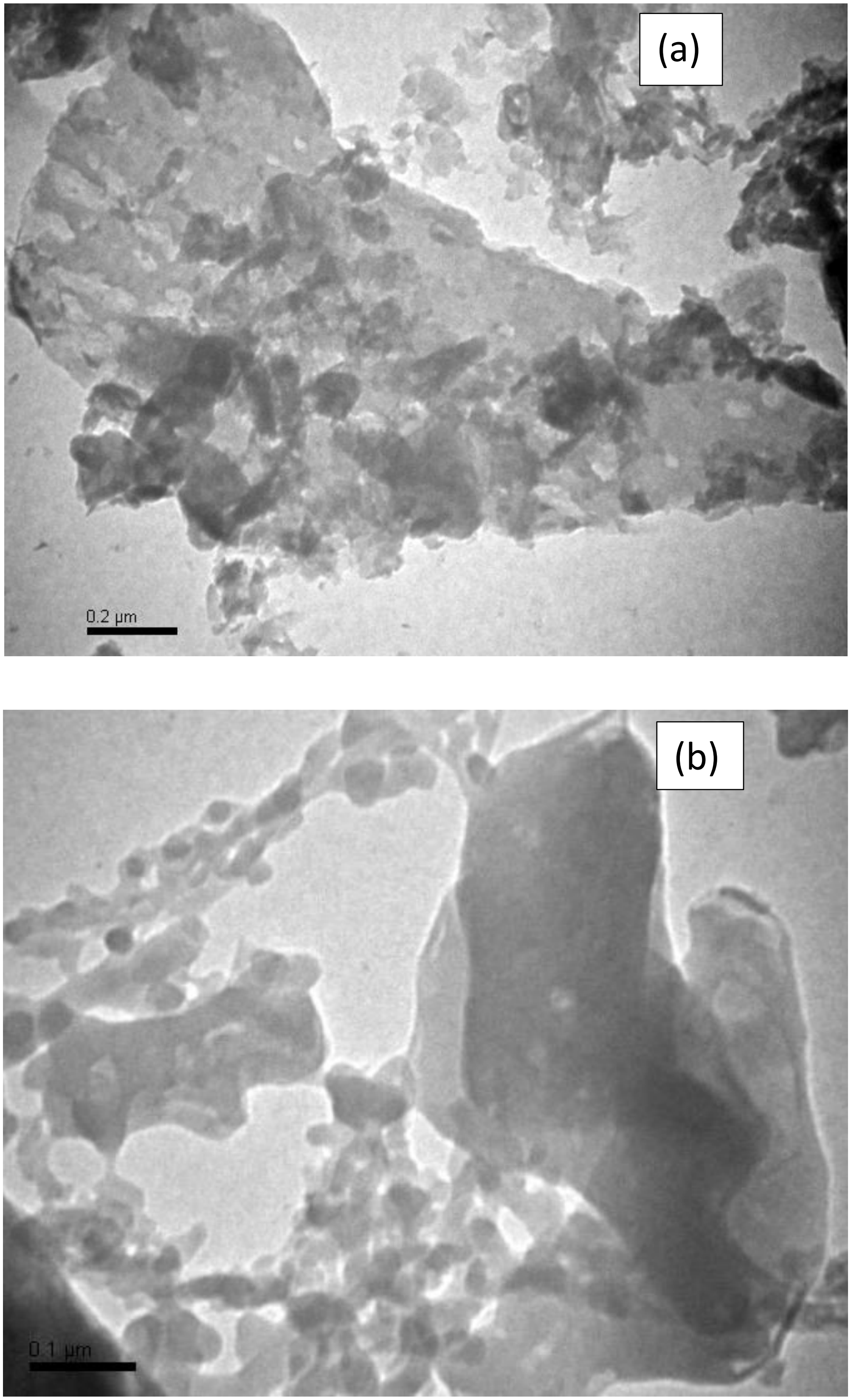
(a)
0.2 µm
(b)
0.1 µm

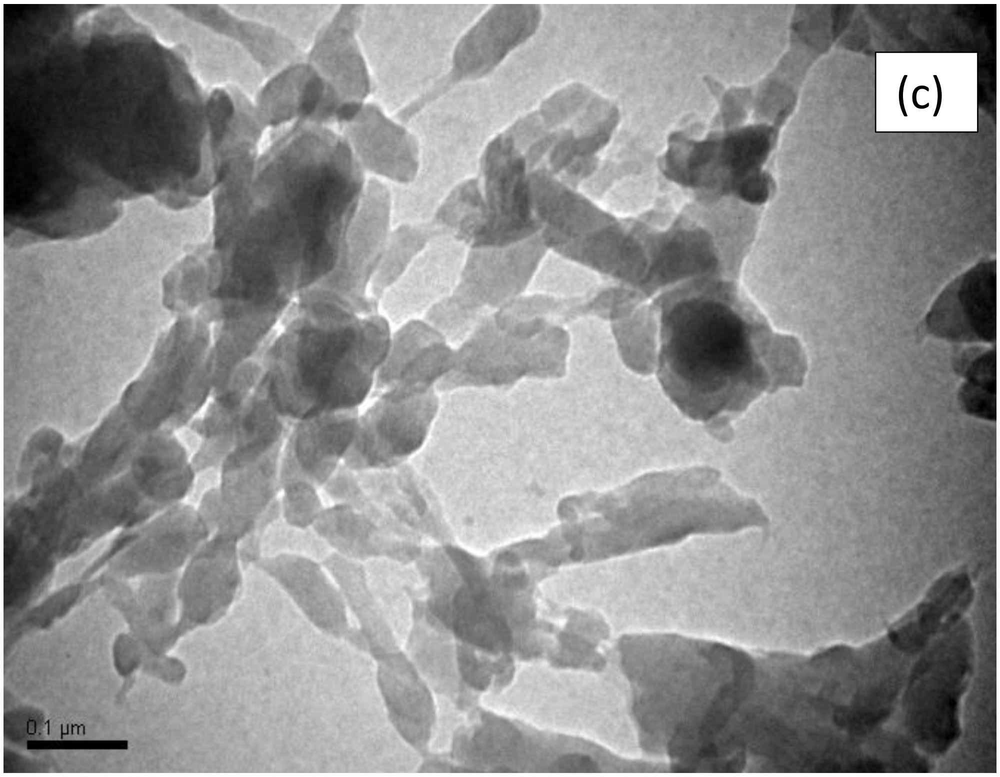

Figure S2. TEM images of (a) g-C_3N_4, (b) Ox-g-C_3N_4, (c) Pani-NF

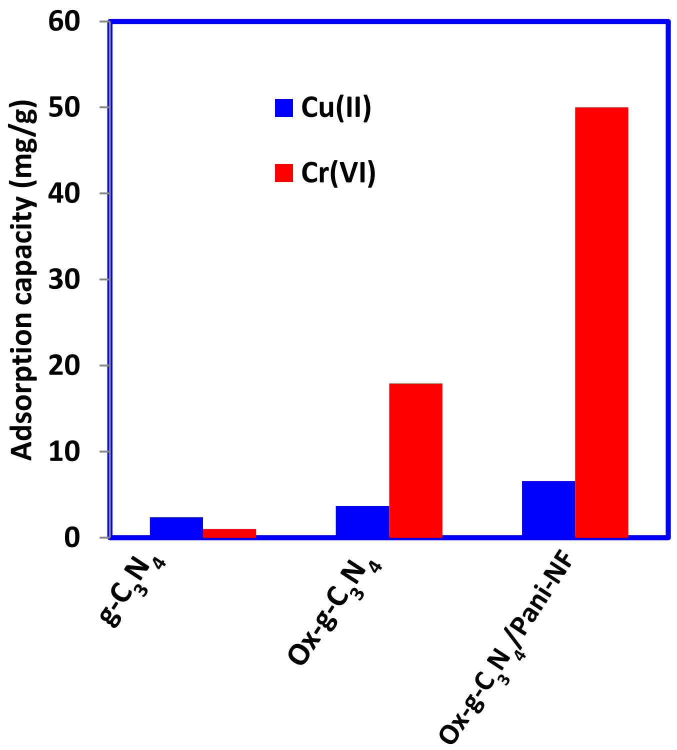

Figure S3. Adsorptive removal of Cu(II) and Cr(VI) by g-C_3N_4, Ox-g-C_3N_4 and Ox-g-C_3N_4/Pani-NF composite (conc.-50 mg/L, vol.-10 ml, time-6h. temp.-30°C)

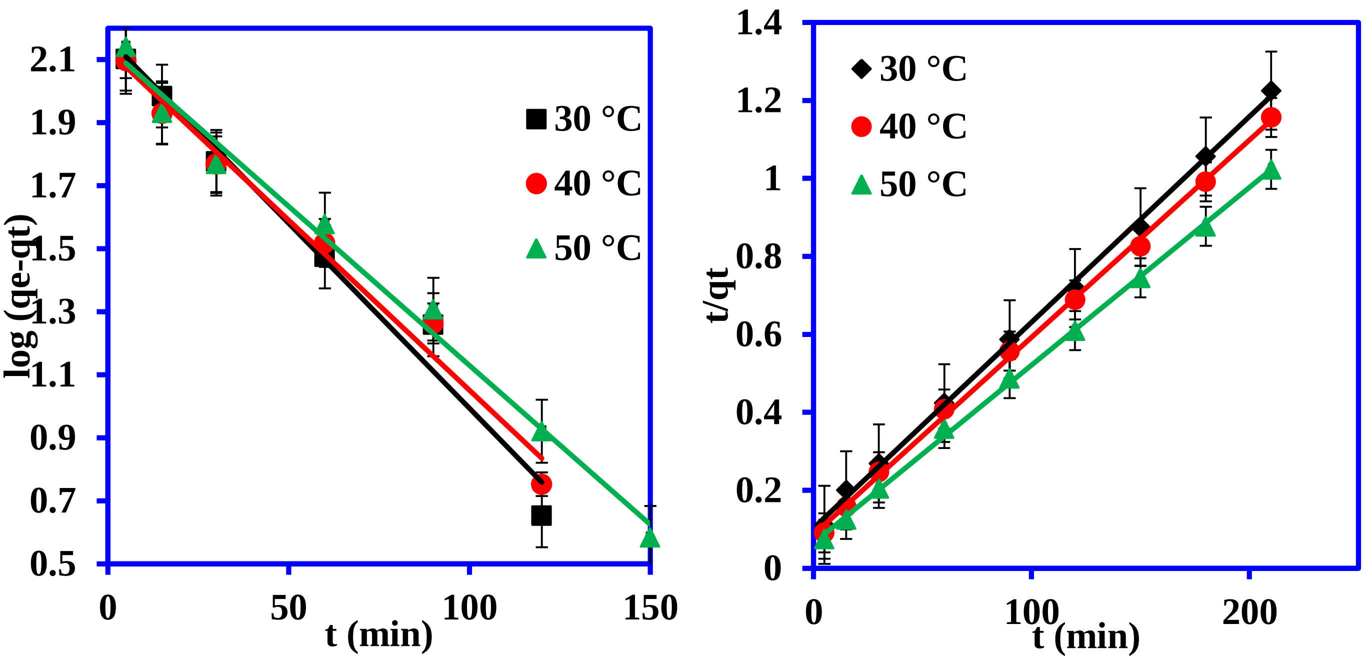

Figure S4. Pseudo-first order (a) and pseudo-second order (b) kinetic plot for Cr(VI) adsorption onto the Ox-g-C_3N_4/Pani-NF composite

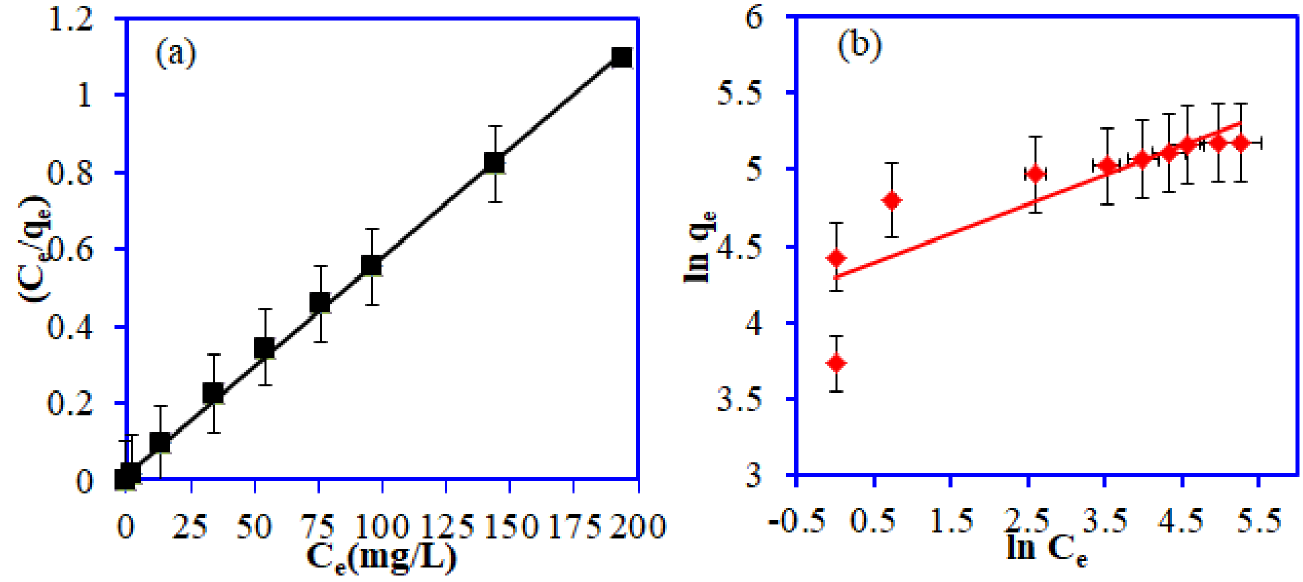

Figure S5. Adsorption isotherm plot for (a) Langmuir and (b) Freundlich model.
